# Resolving the cellular profile of germinal center-analogous microstructures in zebrafish

**DOI:** 10.3389/fimmu.2026.1893026

**Published:** 2026-08-31

**Authors:** Renald James Legaspi, Alison Jessica Lee You Voon, Katharine E. Magor, Brad G. Magor

**Affiliations:** 1 Department of Biological Sciences, University of Alberta, Edmonton, AB, Canada; 2 Li Ka Shing Institute of Virology, University of Alberta, Edmonton, AB, Canada

**Keywords:** affinity maturation, evolution, follicular dendritic cell analogue, melanomacrophage centers, single-cell RNA sequencing, zebrafish spleen

## Abstract

Teleost fish lack canonical germinal centers (GCs); however, they can mount antigen-specific immune responses, raising longstanding questions about how antibody affinity maturation is organized in cold-blooded vertebrates. Melanomacrophage centers (MMCs) have been proposed as GC analogues, yet their cellular composition and immune organization remain poorly defined. To address this, we isolated autofluorescent MMCs from zebrafish spleens and performed single-cell RNA sequencing to characterize their immune and stromal cell populations at high resolution. Our analysis identified centroblast-like and memory-like B cells, helper-like CD4+ T cells, cytotoxic and memory CD8+ T cells, and melanomacrophages with features associated with marginal zone and tingible body macrophages. We also identified a progenitor fibroblastic reticular cell-like population enriched for follicular dendritic cell-associated markers, suggesting a potential role in antigen retention and local immune organization. This was supported by integrating our dataset with publicly available single-cell gene expression data from sharks and frogs. Chemokine expression patterns, including putative CXCR4-CXCL12 interactions, further supported coordinated stromal-lymphocyte organization within MMCs. Together, these findings indicate that zebrafish MMCs contain conserved cellular features associated with affinity maturation, support their role as germinal center-analogues, and provide insight into how germinal centers may have evolved in vertebrates.

## 1 Introduction

In mammals, germinal centers within secondary lymphoid organs are the sites of antibody affinity maturation, the process by which B cells with higher affinity for antigen are selected (1, 2). Within the germinal center, B cells undergo clonal expansion, somatic hypermutation (SHM) of immunoglobulin genes, and selection. In the dark zone (DZ) of the GC, rapidly dividing B cells upregulate activation-induced cytidine deaminase (Aicda), which introduces mutations into immunoglobulin genes, generating a diverse pool of clones with varying affinities (3, 4). These B cells migrate to the light zone (LZ), where follicular dendritic cells (FDCs) display antigens as immune complexes bound to Fc or complement receptors (antigen-Ig-Fc/CR) (5–9). High-affinity B cells process the antigen for MHC class II presentation to cognate T follicular helper cells (Tfh), which deliver survival and differentiation signals (10–13). Selected B cells may undergo class switching (14, 15) and differentiate into plasma cells or memory B cells (15, 16). This iterative process yields up to a 1000-fold increase in antibody affinity compared to the initial response (17, 18).

Fish lack histologically distinct germinal centers (GC) in secondary lymphoid tissues and initial findings of negligible increase in antibody affinity following vaccination, suggested affinity maturation was absent in fish as reviewed previously (19, 20). However, these findings were later attributed to an excess of antigen relative to the number of competing B cells, which impaired selection rather than indicating the absence of affinity maturation (19). Antibody affinity distribution over time demonstrated that rainbow trout antibodies undergo a modest yet measurable ~100-fold increase in affinity after vaccination (20), as also seen with catfish antibodies (21). Similarly, somatic modification, selection of mutations, and affinity maturation have been observed in the IgNAR isotype of nurse sharks (22–24). Previous work (25, 26) identified a functional teleost ortholog of Ig gene mutator, Aicda, supporting the existence of affinity maturation in teleost fish. Using *aicda* expression as a definitive marker of somatic mutation, *aicda*-expressing B cells were found to be near and within aggregates of melanomacrophages (27) in lymphoid tissues of fish, providing a site for an analogous form of antibody affinity maturation. Melanomacrophages accumulate pigments such as lipofuscin, hemosiderin, and melanin, which are byproducts of phagocytosis and the degradation of erythrocytes, leukocytes, and effete cells (28, 29). These pigments confer autofluorescence, allowing convenient identification of melanomacrophages by epifluorescence microscopy. Furthermore in cyprinids including zebrafish, melanomacrophage-lymphoid aggregates are microstructures encapsulated in a reticular cell network, facilitating their isolation (30). These microstructures called melanomacrophage centers (MMCs) have been described in the spleen, kidney, and intestine (28, 31).

Melanomacrophage centers located in the lymphoid tissues of fish have long been suspected as sites of antigen retention and antibody affinity modification in fish (19, 32). Early experiments in fish implicated the melanomacrophages in long-term antigen retention (33, 34), possibly involving antigen-antibody complexes, and nearby B cells suggested they may function as sites of teleost B cell selection like mammalian germinal centers (35). Recent cellular and molecular evidence supports that organized leukocyte aggregates are GC analogues of lower vertebrates, reviewed by Matz and Dooley (36). In sharks, single nuclei RNA sequencing identified lymphoid aggregates containing organized immune populations associated with IgNAR SHM and selection of mutations consistent with affinity maturation (37). In zebrafish, amplification of IgH gene variable regions from isolated MMCs showed evidence of clonal lineages undergoing somatic hypermutation and replacement mutations, suggesting B cell clonal expansion and selection (38). In rainbow trout, proliferating *aicda*^+^ B cells, and T-cell rich regions were found adjacent to MMCs (39). Analysis of the IgM repertoire in melanomacrophage-associated lymphoid aggregates induced by infection suggested these were also sites of B cell clonal expansion. Aicda expression has also been shown in nasal-associated lymphocyte aggregates in trout (40).

Single-cell transcriptomics has become a powerful approach for resolving the cellular composition and organization of germinal-center analogues, as demonstrated by some of the studies described above. Similarly, recent single-cell studies in zebrafish (*Danio rerio*) have generated comprehensive immune atlases of the kidney (41), spleen (42), thymus (43), gut (44), and gill (45), which substantially advanced our understanding of zebrafish immune cell diversity. Recently, Castranova et al. (46) used scRNAsequencing and complementary approaches to characterize the axillary lymphoid organ (ALO), a previously unrecognized secondary lymphoid organ with structural and cellular features resembling mammalian lymph nodes. Interpretation of zebrafish single-cell datasets, however, requires consideration of the teleost-specific whole-genome duplication (Ts3R) (47), which resulted in the retention of numerous duplicated genes, including many immune genes. Many of these paralogs have subsequently diverged through subfunctionalization or neofunctionalization, complicating direct comparisons with mammalian orthologs. Despite this evolutionary complexity, single-cell transcriptomics reveals conserved immune cell populations within lymphoid structures.

Despite these advances, MMCs, which have been proposed as germinal center analogues, have yet to be characterized at a single-cell resolution in teleosts. In this work, using single-cell RNA sequencing, we resolved the cellular composition of zebrafish spleen MMCs, and identified a progenitor fibroblastic reticular cell (pFRC)-like population, which may coordinate with melanomacrophages in antigen handling and B cell selection.

## 2 Materials and methods

### 2.1 Fish maintenance

All fish maintenance conditions and procedures were approved by the University of Alberta Animal Care and Use Committee of the Research Ethics Office, according to guidelines set by the Canadian Council of Animal Care. Fish with sizes ranging from 25 mm - 30 mm and weighing between 0.4 g and 0.5 g were maintained in the UofA Biological fish facilities. Fish were maintained at 26 °C. Only fish with a healthy appearance and no observable clinical signs of disease were used in the experiments. No diagnostic screening for infectious agents was performed.

### 2.2 Hematoxylin and eosin staining and autofluorescence imaging of spleen sections

Spleens were dissected from non-infected zebrafish. Fish were monitored daily for 30 days prior to tissue collection, and no clinical signs of infection were observed during this period. Spleens were fixed overnight in 4% paraformaldehyde (PFA) in phosphate-buffered saline (PBS), then sequentially rinsed in PBS, 25% ethanol, 50% ethanol, and 70% ethanol. Tissues were embedded in paraffin, sectioned at 5 μm, and stained with hematoxylin and eosin (Hematoxylin Gill III, Surgipath 3801542; Eosin, Surgipath 3801602). The middle section of each spleen was imaged using both brightfield and the green fluorescence channel on a Leica DMRXA epifluorescence microscope. Images were analyzed using Fiji.

### 2.3 Lymphoid microstructure sample collection

Spleens were dissected, and lymphoid microstructures were isolated and dissociated into single-cell suspensions. Female fish were used because they consistently yielded higher numbers of cells. For sequencing, two female zebrafish were euthanized using 200 mg/L tricaine methanesulfonate (TMS) (buffered to pH 7 with NaOH) after which they were measured and weighed. Upon fish dissection, the spleen was isolated. The spleen was suspended in GFL-15 filtered media. A total of 6 autofluorescent melanomacrophage centers were identified and isolated from the pooled spleens through microdissection and fluorescence microscopy. Only clearly distinct clusters with strong fluorescence contrasting with the surrounding tissue were isolated. The clusters were disaggregated through a 40 μm mini cell strainer (PluriSelect Mini-Strainer 40 µm mesh) using GFL-15 media as flow through fluid. Disaggregated clusters were resuspended in 0.02% BSA in PBS. We measured the percentage viability of the single-cell suspension at 93.5% using the Trypan Blue exclusion test of cell viability. The total number of cells submitted for single-cell RNA sequencing was 214,000 cells.

### 2.4 Single-cell RNA-sequencing and data analysis

The sample was sent to the University of Alberta Advanced Cell Exploration Core facility to prepare a single-cell library using the Chromium Next GEM Single-cell 3′ Gene Expression v3.1 (Dual Index). The library concentration was at 16.43 μg/µL with an average fragment size of 463 bp. The library was then sequenced at the UofA Molecular Biology Facility using 150 bp paired-end sequencing on the Nextseq550.

The sequence data was initially processed using the Cell Ranger pipeline (10× Genomics) to demultiplex raw base cell files, align them against the NCBI Refseq *Danio rerio* genome (GRCz11; GCF_000002035.6) and ENSEMBL genome release 112, and generate feature-barcode matrices. Subsequent analysis with the Seurat (v5.3.0) R package (48) involved removing low-quality cells based on criteria such as low/excessive unique feature counts (>2500 or <200) and high mitochondrial gene counts (>5%). The QC-filtered data were normalized using the sctransform method for variance stabilization. PCA was performed on the normalized data using the first 30 principal components. Graph-based clustering of cells was conducted across different resolutions (0.1 to 1) using the FindNeighbors and Findclusters functions. Most stable cluster size was identified using single-cell consensus clustering (sc3) stability index using clustree. The resolution with the highest mean and median stability scores across its clusters was selected for further analysis. Clusters with number of cells <1% of the total number cells were considered rare and excluded from further analysis. The most stable cluster/community size determination was performed using sc3 stability index (0 to 1). The 0.4 resolution had the highest mean and median sc3 stability index and was thus used for the subsequent marker-based analysis. The cell populations were visualized as a UMAP using Loupe Browser (v8.0.0). The clusters were annotated using both marker-based analysis and through performing STRING analysis (49) on differentially expressed genes to identify enriched pathways as an indication of cell type function.

### 2.5 Agnostic doublet scoring

Doublet detection was performed using DoubletFinder (v2.0) (50) on raw expression matrix to retain all cells, including those with unusually high or low feature counts, and predicted doublet barcodes were annotated. To infer the most likely lineage contributions for each doublet, we used the cluster marker genes identified in the preceding differential expression analysis. Module scores were calculated with Seurat AddModuleScore using the normalized sctransform data and the top 100 marker genes per cluster. For each predicted doublet, the two highest module scores (Top1 and Top2) were interpreted as the dominant lineage signatures contributing to that doublet transcriptomic profile.

### 2.6 Cross-species projection and gene set enrichment analysis

Conservation of immune transcriptional programs was assessed using complementary cross-species projection and gene set enrichment approaches. Differentially expressed genes for zebrafish clusters S3 and S7 were identified using Seurat (v5.3.0) with SCT-normalized data (Wilcoxon rank-sum test; min.pct ≥ 0.20; log_2_FC ≥ 0.25; positive markers only), excluding ribosomal and mitochondrial genes. The top 200 markers were retained for cross-species projection.

Shark single-nucleus RNA-sequencing data (37) were processed using standard Seurat workflow, including normalization, variable feature selection (n = 2,000), scaling, principal component analysis, UMAP (dims 1-30), and graph-based clustering (resolution = 0.5). Zebrafish markers were intersected with shark gene symbols, and shared genes were used to compute per-cell module scores using AddModuleScore. Shark clusters were ranked by mean module score to nominate candidate homologous populations. Marker genes from the top-ranked shark cluster were identified (Wilcoxon rank-sum test; min.pct ≥ 0.20; log_2_FC ≥ 0.25), and overlap with lineage-defining gene sets from the original shark study was quantified using fractional overlap and hypergeometric enrichment testing.

In parallel, conservation of mammalian immune programs was evaluated using cross-species gene set enrichment analysis. Mouse gene sets (51) were converted from *Mus musculus* to *Danio rerio* orthologs using orthogene (v1.17.2), retaining genes present in the zebrafish SCT-normalized matrix. When multiple zebrafish paralogs mapped to a single mouse gene, per-cell median expression was calculated. Per-cell enrichment scores were computed using AUCell as implemented in escape (v2.4.0), and median scores were calculated per cluster. Pairwise differences between S3 and S7 were evaluated using Wilcoxon rank-sum tests with Benjamini-Hochberg correction, and effect sizes were estimated using Cliff’s delta (effsize v0.8.1).

## 3 Results

### 3.1 Single-cell RNA sequencing shows diverse cell heterogeneity in zebrafish spleen melanomacrophage clusters

**Figure 1:**
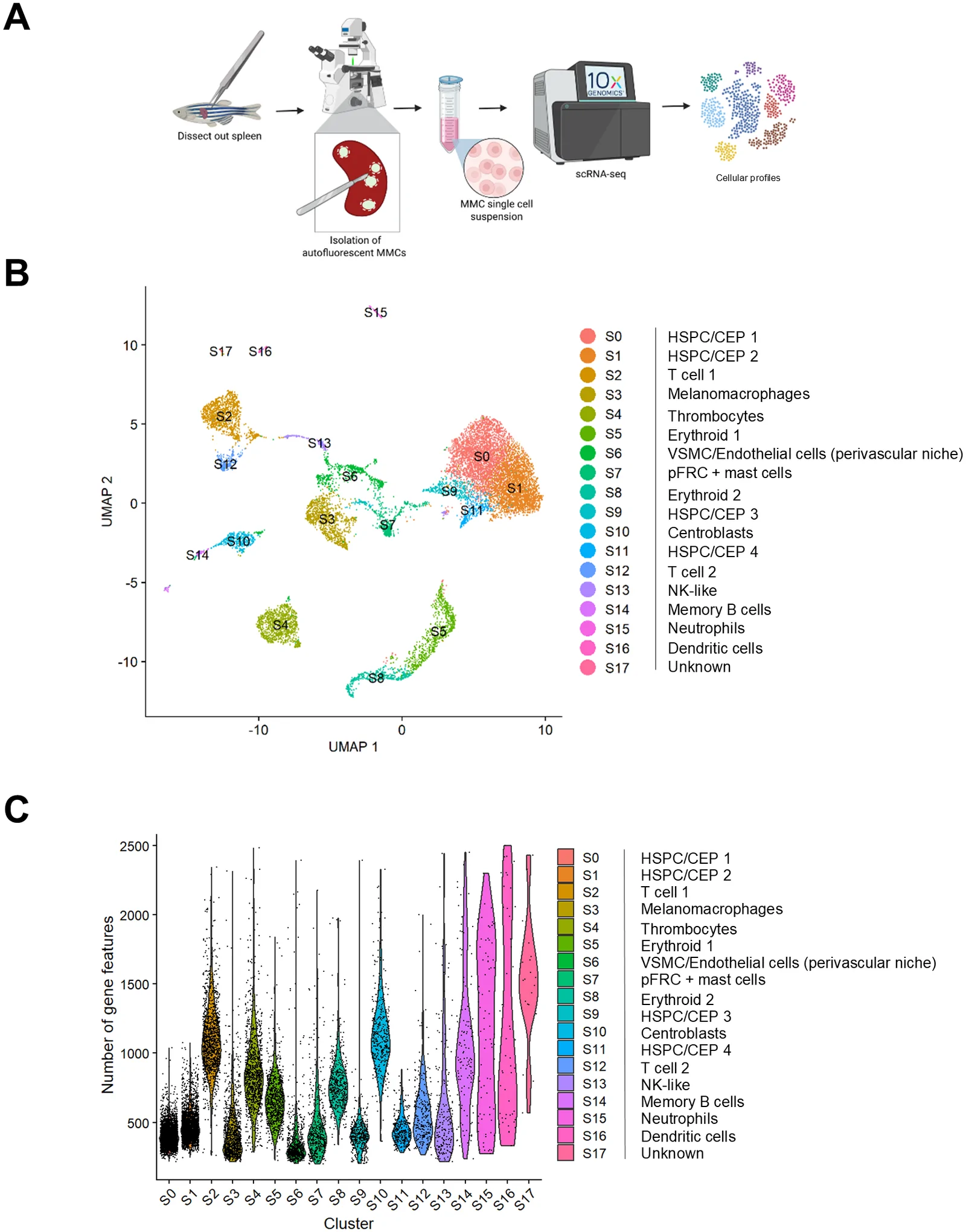
Single-cell RNA sequencing shows diverse cell heterogeneity in zebrafish spleen melanomacrophage clusters. **(A)** Experimental workflow for single-cell RNA sequencing of zebrafish melanomacrophage centers (MMCs). **(B)** The UMAP obtained from our dataset and visualized using Cloupe Browser from 10X Genomics. **(C)** Violin plots representing the distribution of gene features for each cluster at resolution 0.4. The clusters S15, S16 and S17 were removed from the subsequent marker-based analysis owing to their broad distribution of features, suggesting heterogeneous populations. HSPC, hematopoietic stem and progenitor cells; CEP, common erythroid progenitor; VSMC, vascular smooth muscle cells; pFRC, progenitor fibroblastic reticular cells; NK, natural killer cells.

To define cell types within zebrafish MMCs under unchallenged conditions, we isolated and pooled MMCs from spleens and performed single-cell RNA sequencing (scRNA-seq) (**Figure 1A**). Subclustering at resolution 0.4 resulted in the highest mean and median cluster stability, with 17 clusters from 13,632 cells. We identified four hematopoietic stem and progenitor cell, and common erythroid progenitor (HSPC/CEP) clusters (S0, S1, S9, S11), two T cell clusters (S2, S12), two erythroid cell clusters (S5, S8), two B cell clusters (S10, S14), melanomacrophages (S3), thrombocytes (S4), endothelial cells and vascular smooth muscle cells (VSMCs) (S6), progenitor fibroblastic reticular cell-like (pFRC) and mast cells (S7), NK-like cells (S13), neutrophils (S15), and dendritic cells (S16) (**Figure 1B**). To assess the resolution of cell types, we profiled the variability of gene expression between cells within a cluster, revealing that subclusters 0–14 showed restricted gene expression profiles (**Figure 1C**). The smallest clusters (S15-S17) exhibited the highest intra-cluster variability in gene expression, with S17 remaining unidentified (**Figure 1C**). Due to this variability, these clusters were excluded from downstream comparative analyses across cell types.

To identify cell types largely restricted to the MMC, we integrated our dataset with a single-cell transcriptome of whole zebrafish spleen injected with PBS (42). The integrated dataset contained 27 clusters. We then remapped barcodes from the MMC onto our 17-cluster UMAP to help assign cell types to the 27 integrated clusters. This analysis showed that the integrated clusters 0, 8, 10, 12, 14, 19, 21, and 26 were enriched for MMC-derived cells relative to PBS-spleen cells (**Supplementary Figure 1A**). These clusters corresponded to HSPC/CEPs, erythroid cells, pFRCs, endothelial and vascular smooth muscle cells, memory B cells, and two T cell subclusters (**Figure 1B**; **Supplementary Figure 1B**). Melanomacrophage (integrated cluster 5), thrombocyte (integrated cluster 4), and VSMC/endothelial cell (integrated cluster 14) integrated clusters also contained substantial contributions from MMC-derived cells, indicating that these populations are enriched within the MMC despite also being distributed throughout the spleen (**Supplementary Figures 1A, B**).

### 3.2 Zebrafish MMC B cell subpopulations include centroblasts and memory B-like cells

**Figure 2:**
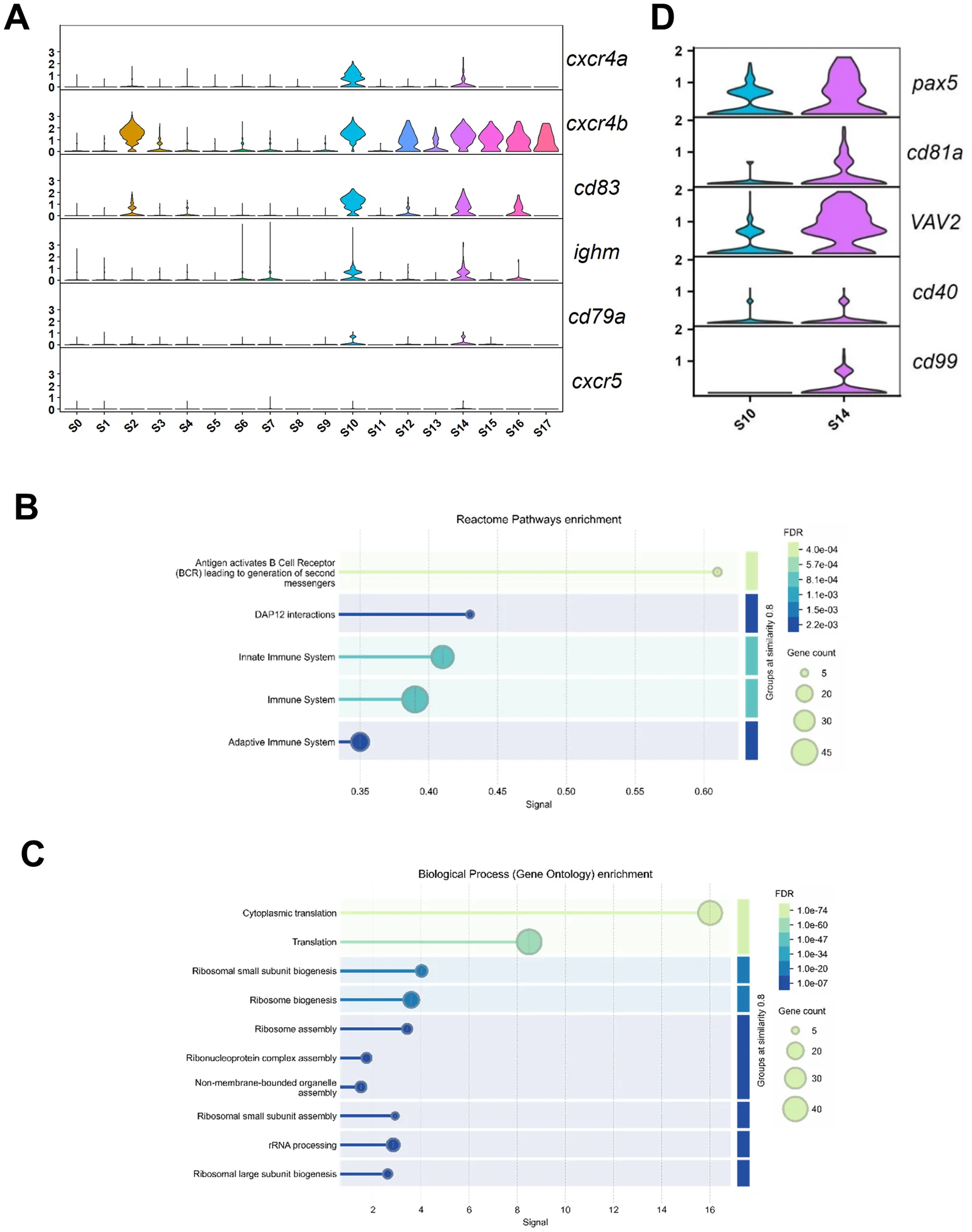
Zebrafish MMC B cell subpopulations include centroblasts and memory B-like cells. **(A)** Violin plots showing the differential expression of the centroblasts and B cell markers in cluster S10, identified as centroblasts. **(B)** STRING most enriched pathways of S10, featuring BCR activation and adaptive immune function, aligned with the identification of S10 as centroblasts. **(C)** STRING biological processes enrichment of S10, showing the differentially expressed S10 genes involved in various transcription and translation processes. This aligns with the high metabolic activity of the centroblasts during clonal expansion. **(D)** Violin plots representing the higher expression of memory B-cell-like markers in S14 compared to S10.

To identify B cell subtypes in zebrafish spleen MMCs, we performed differential gene expression analysis of key B cell markers between the two clusters followed by STRING pathway enrichment analysis. Comparison of gene expression across all subclusters revealed S10 and S14 had the highest expression of B cell markers (**Figure 2A**). Subcluster S10 showed the highest expression of *cxcr4a* and *cd83* (**Figure 2A**), markers associated with germinal center (GC) centroblast dynamics. In mammals, proliferating centroblasts in the GC dark zone (DZ) express CXCR4 and localize in response to CXCL12-expressing reticular cells (CRCs), while light zone (LZ) B cells re-enter the DZ proliferation (DZp) area to divide, during which they also express CD83 (52). Consistent with this, STRING analysis indicated that S10 is enriched in the Reactome pathway for antigen-activated BCR signaling (**Figure 2B**), which aligns with differential expression of *ighm* and *cd79a*, components of the BCR, in S10 (**Figure 2A**). Pairwise comparison between S10 and S14 further revealed enrichment of translation-related pathways in S10, suggesting increased proliferative activity (**Figure 2C**) and strengthening our identification of S10 as centroblast-like cells.

S14 showed higher expression of *pax5*, *cd81a*, *vav2*, *cd40*, and *cd99* than S10 (**Figure 2D**); thus, we classified it as a memory B cell population. Memory B cells maintain their identity through the transcriptional repressor PAX5, which must be inactivated for their terminal differentiation into plasma cells (53). Both centrocytes and memory B cells express lower levels of CXCR4 compared to centroblasts (54, 55). In mice, the CD19/CD21 complex, a crucial coreceptor in B cell responses to T cell-dependent antigens, collaborates with the BCR to lower the activation threshold (56). CD19 serves as a membrane adaptor for the BCR, enhancing Lyn kinase activity and recruiting Vav, which activates MAPKs and PI3K. In mature B cells, CD81 regulates CD19 expression and partners with CD19 and CD21 to further reduce the activation threshold (57). Additionally, cross-linking of CD40 on B cells by CD40L on T cells prevents apoptosis and promotes differentiation into memory B cells or plasma cells (58). Memory T and B cells in reactive lymph nodes exhibit high CD99 expression, with IgG+ B cells showing significantly higher levels than IgM+ B cells (59).

### 3.3 Zebrafish MMC T cell subpopulations include cytotoxic, memory, and Th2-like T cells

**Figure 3:**
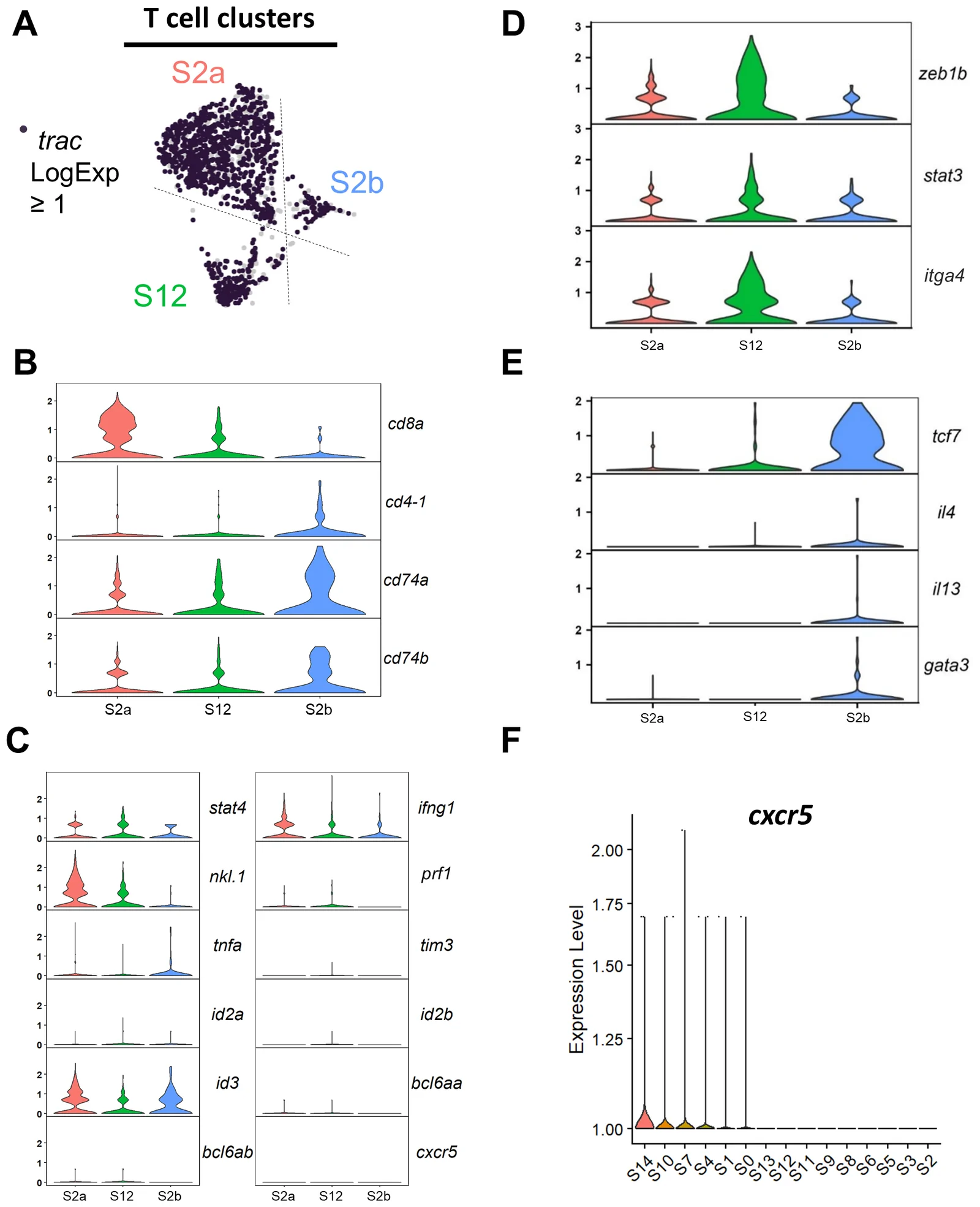
Zebrafish MMC T cell subpopulations include cytotoxic, memory, and Th2-like T cells. **(A)** UMAP visualization of T cell subclusters S2a, S2b and S12 at resolution 0.5 showing log-normalized expression of TCR alpha constant gene (*trac*) ≥ 1. **(B)** Violin plots showing higher differential expression *cd8a* in clusters S2a and S12 identified as CD8+ T cells, and CD4+ T markers (*cd4-1*, *cd74a/b*) in cluster S2b. **(C)** Violin plots showing higher differential expression of Tc1-related genes *stat4*, *ifng1*, *nkl1*, *PRF1* and *tnfa*, and Tfc-related genes *id3*, *bcl6ab*, *bcl6aa* in cluster S2. **(D)** Violin plots showing higher differential expression of memory markers *zeb1b*, *stat3*, and *itga4* in cluster S12, identified as memory CD8+ T-cells. **(E)** Violin plots showing highest expression of *tcf7*, *gata3* (Th2 differentiation markers) and *il4*, *il13* (Th2 cytokines) in cluster S2b. **(F)** Sparse expression of *cxcr5* across the dataset.

To assess T cell clustering, we analyzed the data at different resolutions. At 0.4 resolution, T cells form two sub-clusters, but at higher resolutions (0.5 and above), S2 splits into two subsets (**Figure 3A**). The mean SC3 stability at 0.4 and 0.5 resolutions was similar (0.603 vs. 0.604), suggesting stable clustering. At 0.5 resolution, we identified three T cell subsets (S2a, S2b, S12). S2a and S12, with high *cd8a* expression and *trac* (TCR alpha constant), are identified as CD8+ T cells (**Figures 3A, B**), while S2b is identified as CD4+ T cells based on the high expression of *cd4-1*, *cd74a*, and *cd74b* (**Figure 3B**). CD8+ T cells are MHC-I restricted cytotoxic effectors, while CD4+ T cells are MHC-II restricted helpers (60). In humans, CD4+ T cells express intracellular CD74, a MIF receptor, which increases upon activation (61).

To define CD8+ T cell subsets, we examined genes associated with their differentiation and function. Based on higher expression of cytotoxic effector cell (Tc1)-related genes (*stat4*, *ifng1*, *nkl1*, *PRF1*, *tnfa*), we classified S2a as a Tc1 population (**Figure 3C**). As characterized primarily in mammals, Tc1 cells produce perforins (PRF1), IFN-γ, and TNF-α (62). They also upregulate STAT4 to enhance IFN-γ production (63). Cytotoxic T cells also secrete antimicrobial peptides like NK-lysin (64). CD8+ T cells develop in the thymus, differentiate into, and form memory cells for rapid future responses.

To assess memory CD8+ T cells, we investigated markers linked to their differentiation. S12 showed increased expression of memory markers, including *zeb1b*, *stat3*, and *itga4* (**Figure 3D**). As shown in mice, memory T cell formation is regulated by factors like Zeb1 (65) and STAT3, which are essential for memory (66). Indeed, the memory nature of S12 CD8+ cells was reinforced through its genetic signature resemblance to cxcr5+ T follicular cells (Tfc) that show high expression of *id3*, *bcl6ab*, *bcl6aa* and low expression of *id2a/b* and *tim3* (**Figure 3C**). Notably, unlike cxcr5+ Tfc cells, cluster S12 cells did not express *cxcr5*, suggesting that *cxcr5* is not part of the T-cell homing strategy in teleost lymphoid organs.

For CD4+ helper T cells, we classified S2b as Th2-like based on the upregulation of Th2 cytokine genes (*il4*, *il13*) and transcription factor *gata3 (*62). S2b also expressed *tcf7*, important for Th2 differentiation and inhibiting Th1 development (67) (**Figure 3E**). We then checked for *cxcr5* expression, which is important for T follicular helper cells to migrate to B cell follicles (68). However, *cxcr5* was not expressed in any T cell subset, although sparse expression was found in other clusters including B cells (**Figure 3F**).

### 3.4 Zebrafish melanomacrophages have features of multiple different macrophage subtypes

**Figure 4:**
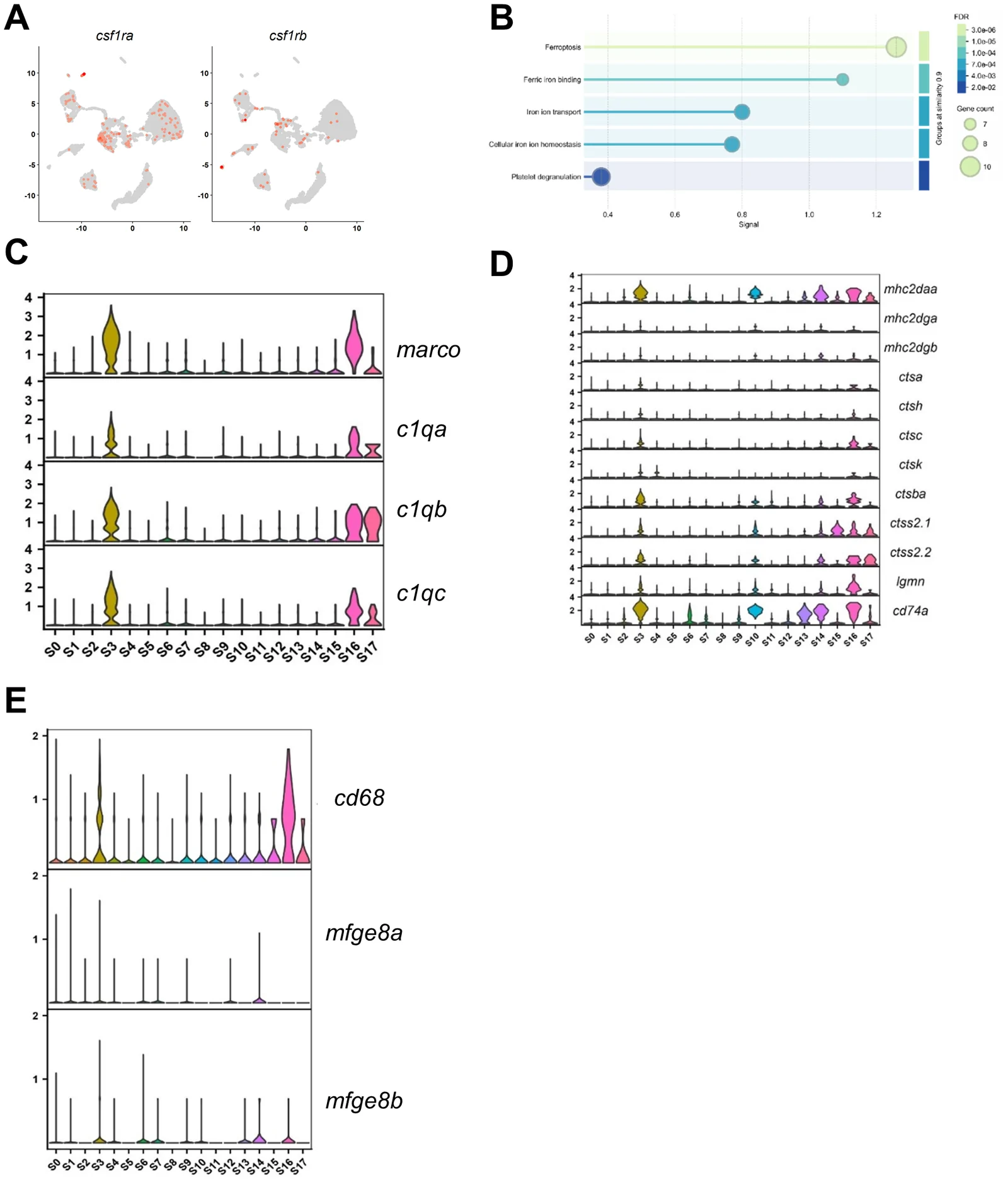
Zebrafish melanomacrophages have features of multiple different macrophage subtypes. **(A)** UMAP plots showing highest expression of *csf1ra* and *csf1rb* in S3 suggests it is the melanomacrophage population in our dataset. **(B)** STRING enrichment analysis of cluster S3 revealing upregulation of several genes associated with iron transport and processing, which are implicated in accumulation of lipofuscin and hemosiderin. **(C)** Violin plots showing high differential expression of *marco* (scavenger receptor), and *c1qa/b/c* (genes of the complement pathway) in cluster S3. These are markers of marginal zone macrophages (MZMs). **(D)** Unlike MZMs, S3 shows high expression of MHC class II-related genes together with antigen processing-associated genes including cathepsins and legumain (*lgmn*). **(E)** Violin plots showing differential expression of genes associated with tingible body macrophages, specifically apoptotic debris clearance, in cluster S3.

Zebrafish macrophages in the spleen MMC consist of a single population (S3) displaying features of teleost melanomacrophages (MM), mammalian marginal zone macrophages (MZMs), and mammalian tingible body macrophages (TBMs) (**Figure 4**). In mammals, macrophages are regionally specialized: red pulp macrophages clear aged erythrocytes and process iron, MZMs capture blood-borne antigens and regulate B cell retention, while metallophilic macrophages (MMMs) perform immune surveillance.

To determine if S3 macrophages exhibit features of melanomacrophages (MMs), we analyzed their gene expression profile and pathway enrichment. In fish, macrophages in melanomacrophage centers (MMCs) contribute to phagocytosis, including erythrophagocytosis, and express colony-stimulating factor 1 receptor (CSF1-R) (69). Given its high cumulative gene expression of *csf1ra* and *csf1rb* and its relatively large proportion (8%) in the spleen MMC, we identified S3 as the primary macrophage population in MMCs (**Figure 4A**).

S3 was enriched in iron processing and ferroptosis-related pathways, indicating its potential for erythrophagocytosis and accumulation of autofluorescent pigments like melanin, lipofuscin, and hemosiderin (**Figure 4B**). MMs are known for erythrophagocytosis, which induces ferroptosis in macrophages (70). Ferroptosis, marked by lipid peroxidation, causes cell membrane damage and the formation of lipofuscin, a pigment resulting from incomplete degradation of oxidized protein-lipid complexes (71). Erythrophagocytosis also leads to iron overload and hemosiderin accumulation (72).

S3 showed high expression of the macrophage scavenger receptor MARCO and genes associated with non-classical complement pathways such as C1q (**Figure 4C**). MARCO helps bind and ingest unopsonized particles while C1q enhances phagocytosis independent of the classical complement pathway (73). Interestingly, MZMs also express MARCO and interact with C1q to enhance apoptotic cell clearance (74–76). MZMs do not express MHC class II molecules that aid in antigen presentation to B cells (77). In contrast, S3 showed high expression of MHC class II-related genes and several cathepsin family members (**Figure 4D**). TBMs express MHC class II and play a crucial role in antigen presentation and T cell interactions in germinal centers (78). Also, S3 macrophages share significant similarities with TBMs, including high expression of CD68 and genes related to milk fat globule-EGF factor 8 (MFGE8), which is critical for apoptotic cell clearance (79, 80) (**Figure 4E**). This suggests that S3 macrophages may function similarly to mammalian TBMs, contributing to apoptotic cell clearance and immune regulation.

Given these findings, we propose that the S3 cells are the melanomacrophages. S3 cells appear to integrate features of both MZMs and TBMs, as they exhibit characteristics of both antigen-capture and apoptotic cell-clearance functions, which are typically seen in organized B cell follicles.

### 3.5 Zebrafish MMC have perivascular analogues of mammalian vascular smooth muscle cells, endothelial cells, and follicular dendritic cell precursors

**Figure 5:**
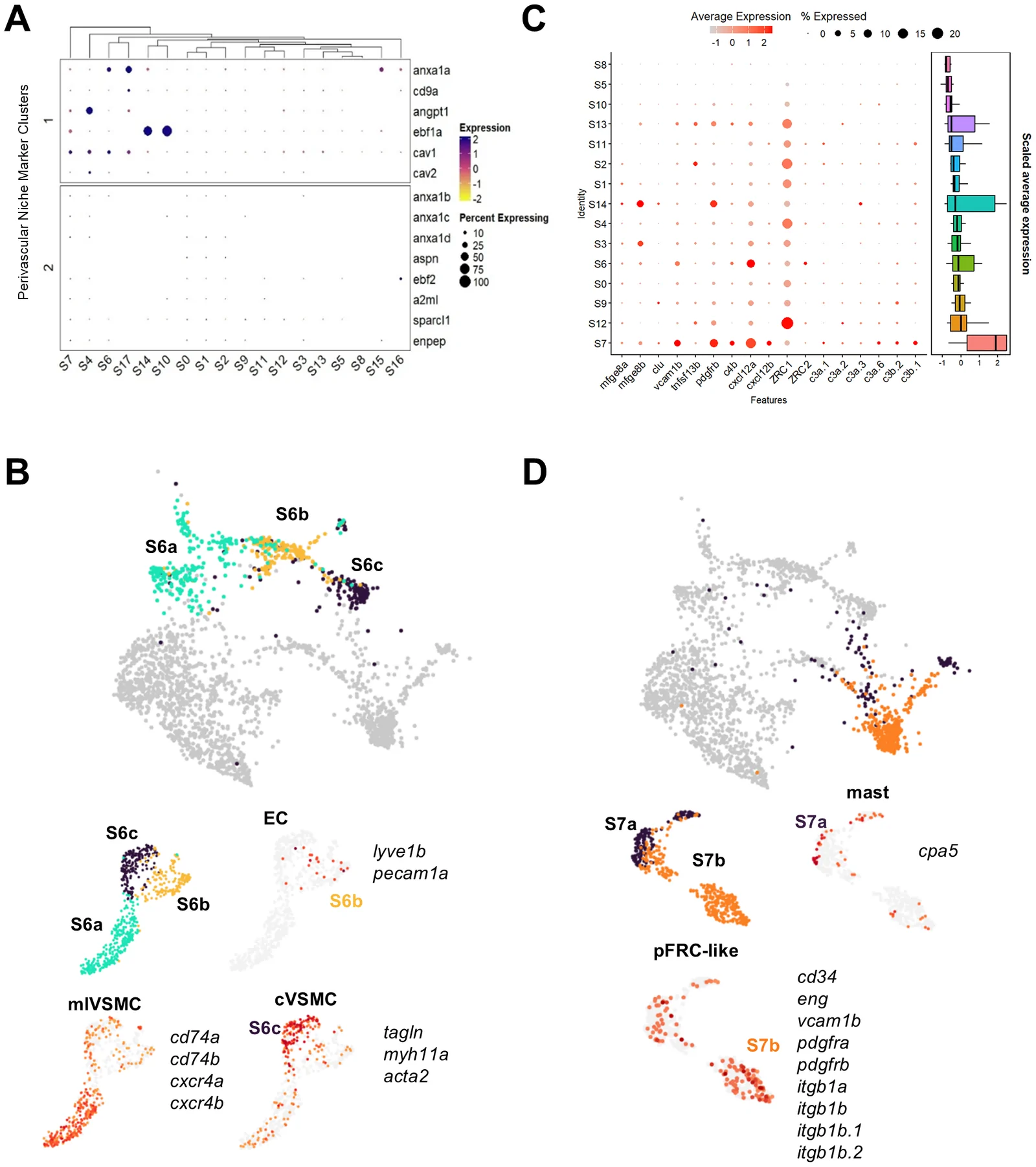
Zebrafish MMC have perivascular analogues of mammalian vascular smooth muscle cells, endothelial cells, and follicular dendritic cell precursors. **(A)** Dot plot of perivascular niche-associated genes clustered into high (1) and low (2) expression. S7, S4 and S6 show the highest average expression of the highly expressed gene cluster. **(B)** Sub-clustering of cluster S6 into 3 subclusters, namely S6a (macrophage-like VSMCs), S6b (endothelial cells) and S6c (contractile VSMCs). **(C)** Dot plot showing the highest cumulative expression of the FDC-associated genes in cluster S7 across clusters S0 - S14. **(D)** Sub-clustering of cluster S7 into 2 subclusters, namely S7a (mast cells) and S7b (pre-follicular reticular cells).

To identify analogues of mammalian splenic fibroblastic reticular cells (FRCs) and their precursors, we analyzed the expression of markers associated with the perivascular niche, the proposed origin site of these cells in the mammalian spleen (81–83). Clusters S7, S4, and S6 exhibited the highest cumulative expression of perivascular niche markers (**Figure 5A**). Further subclustering of S6 revealed two vascular smooth muscle cell (VSMC)-like populations (S6a and S6c), along with an endothelial population expressing *lyve1b* and *pecam1a* (**Figure 5B**) (84). S6c showed high expression of contractile VSMC markers (tagln, myh11a, acta2), while S6a expressed both contractile markers and genes characteristic of macrophage-like VSMCs (85).

To determine whether these perivascular populations define a stromal-immune niche, we analyzed putative cell doublets based on combined lineage signatures. This revealed a dominant and reproducible pairing of S6 with B cells (S10, S14), T cells (S2), and MMs (S3) (**Supplementary Figure 2A**). Top1-Top2 score distributions supported these interactions, where narrow gaps indicated true co-encapsulation or tightly associated cell pairs, while wider gaps suggested contributions from ambient RNA or apoptotic debris (**Supplementary Figure 2B**). Together, these findings support a vascular-associated role for S6 and suggest that it represents a blood vessel population adjacent to MMC.

To identify potential follicular dendritic cell (FDC) analogues in zebrafish, we analyzed the expression of orthologues of key mammalian FDC markers, including *mfge8* (FDC-M1), *clu* (clusterin), *vcam1*, *tnfsf13b* (BAFF), *c4* (FDC-M2), and *cxcl12* across all clusters (86–89). We also considered the expression of complement C3 which has been shown to be produced from human FDCs (90). Other prominent markers of FDC include CR1 and CR2. While the orthologues of CR1/2 have not been conclusively defined yet, the syntenic conservation of ZRC1 and ZRC2 in zebrafish with CR1 and CR2 in mammals could be indicative of CR1/2 ancestry. As such, we also examined the expression of ZRC1/2 across our dataset. Cluster S7 showed the highest cumulative expression of FDC markers, complement C3 and ZRC1/2 (**Figure 5C**), positioning it as an FDC analogue. However, because zebrafish lack the lymphotoxin beta receptor (LTBR) and the LT/LTβR signaling pathway (91), which is essential for the maturation of fibroblastic reticular cell progenitors into specialized subsets such as FDCs, marginal reticular cells (MRCs), and T zone reticular cells (TRCs) using *in vivo* mouse models (82), we hypothesize that S7 more closely resembles a perivascular FDC precursor (preFDC) rather than a fully differentiated FDC. To investigate this, subclustering of S7 revealed a small population of mast cells (S7a), characterized by cpa5 expression, and a progenitor FRC (pFRC)-like intermediate population (S7b) (**Figure 5D**). S7b showed high expression of *cd34*, cd140a/b (*pdgfra/b*), and cd29 (*itgb1*).

### 3.6 Zebrafish MMC show dominant transcriptional signatures of CXCR4-CXCL12 and CCR9-CCL25

**Figure 6:**
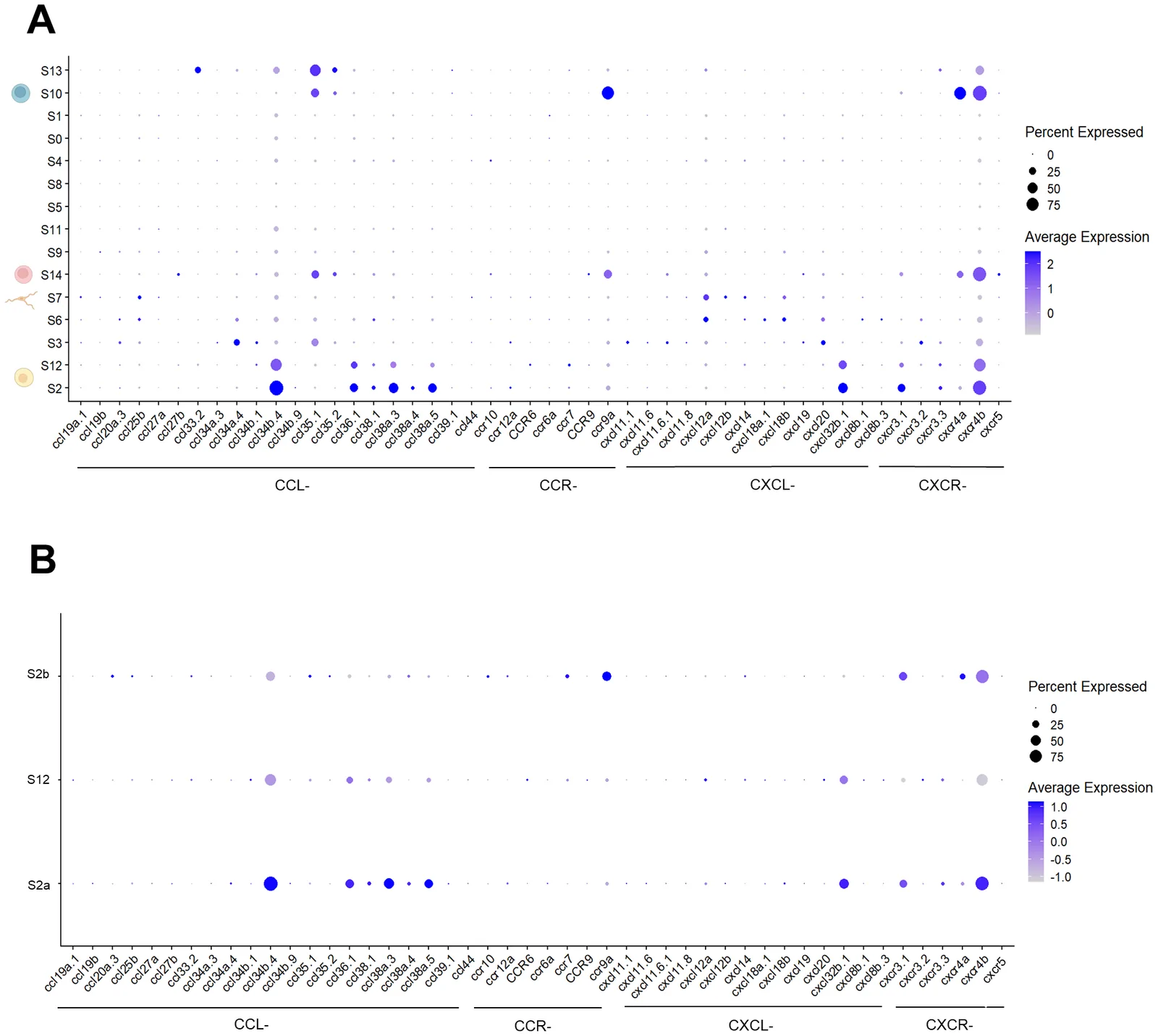
Zebrafish MMC shows dominant transcriptional signatures of CXCR4-CXCL12 and CCR9-CCL25. **(A)** Dot plot showing the expression of chemokine ligands and receptors across clusters S0-S14. We note the highest differential expression of *ccr9a* in cluster S10 (centroblasts), and its ligand *ccl25b*, which is highly expressed by S7 (pFRCs). We also note the expression of *cxcl12a/b* by S6 and S7 and expression of *cxcr4a/b* by B-cells (S10 and S14) and T-cells (S2 and S12) which suggests a potential *cxcr4-cxcl12* gradient in the zebrafish spleen MMC. **(B)** Dot plot showing the expression of chemokine ligands and receptors of the three T-cell subclusters S2a, S2b and S12. Subcluster S2b (Th2 cells) has the highest differential expression of *ccr9a* which we suggest could allow its potential migration to *ccl25b*-expressing S7 (FDC analogue) cells. Both subclusters S2a (Tc1 cells) and S2b (Th2 cells) have a differentially high expression of *cxcr4b*, with subcluster S2b also having high differential expression of *cxcr4a*, which suggests migration of these cell types to *cxcl12*-expressing S7 cells (pFRCs).

To investigate chemokine signaling activity in the zebrafish spleen MMC, we examined the expression of ligand–receptor pairs with focus on B and T cells. Among the B cell subclusters, S10 and S14 expressed high levels of *cxcr4a* and *cxcr4b*, indicating potential responsiveness to CXCL12 signaling (**Figure 6A**). The chemokine receptor *ccr9a* was also enriched in these clusters, particularly in S10. All T cell subsets exhibited a similar pattern of *cxcr4a/b* expression, indicating that responsiveness to CXCL12 may be a shared feature across zebrafish T cells. However, *ccr9a* expression was restricted to the S2b cluster, which we identified as a Th2-like T cell subset. (**Figure 6B**)

Notably, cluster S7, previously identified as a pFRC-like population, showed the highest expression of the chemokine ligand genes, *cxcl12a* and *ccl25b* (**Figure 6A**). This suggests that pFRC-like cells may contribute to the MMC chemokine landscape by producing signals that attract both B and T cells.

Although spatial context cannot be determined from transcriptomic data alone, the coordinated expression of *cxcl12a* and *ccl25b* in pFRC-like cells, along with *cxcr4a* and *ccr9a* in lymphocyte subsets, suggests the presence of chemokine signaling axes within the zebrafish MMC transcriptome.

### 3.7 Zebrafish MMC melanomacrophage (S3) and pFRC-like (S7) signatures map to conserved myeloid and stromal lineages across vertebrates

To evaluate the conservation of zebrafish GC-analogous populations across vertebrates, we compared the transcriptional signatures of MMs (S3; 1,090 cells) and pFRC-like cells (S7b; 324 cells) with two independent shark spleen single-nucleus RNA-sequencing datasets (GSM7325057, s1; GSM7325058, s2) (37). Within the zebrafish dataset, differential expression analysis against all other clusters identified 148 S3 marker genes and 93 S7b marker genes after excluding ribosomal and mitochondrial genes (**Supplementary Table 1**). We then performed clustering separately for each shark dataset and identified marker genes for every shark cluster. Cross-species comparison of zebrafish and shark marker genes identified 28 shared genes for S3 (18.9% retention) and 15 shared genes for S7b (16.1% retention) (**Supplementary Tables 1**, **2**). Using these shared genes, we calculated the average enrichment score for each shark cluster and ranked them to identify the closest transcriptional matches to zebrafish S3 and S7b (**Supplementary Table 3**). For shark clusters with the highest enrichment scores, we examined their marker genes and compared them with the cell-type marker sets used for annotation in the original shark study.

We observed the strongest enrichment of the zebrafish S3 signature in cluster 1 of shark dataset s1 (mean = 0.261; 672 cells) and cluster 16 of shark dataset s2 (mean = 0.297; 154 cells) (**Supplementary Table 3**). To infer the identities of these enriched clusters, we compared their marker genes with the annotated cell populations defined in the original shark study. Cluster 1 of s1 showed moderate overlap with the naïve splenic B-cell (B1; overlap fraction = 0.47) and plasmablast (B5; 0.44) populations. In contrast, cluster 16 of s2 showed the greatest overlap with the myeloid population My2 (0.83), which was characterized in the original study by relatively high expression of lilra5, ass1, and cd9 compared with the other myeloid populations (My1 and My3) (**Supplementary Table 4**).

In contrast, the zebrafish S7 signature was most strongly enriched in cluster 7 of s1 (mean = 0.344; 306 cells) and cluster 11 of s2 (mean = 0.287; 260 cells) (**Supplementary Table 3**). Comparison of the marker genes from these enriched clusters with the annotated shark cell populations showed the greatest overlap with the stromal lineage Str1 in both datasets (s1 = 0.96; s2 = 0.78) (**Supplementary Table 4**). Together, these analyses indicate that the S7 signature consistently aligned with the shark stromal lineage Str1 across both datasets, whereas the S3 signature was most strongly associated with the myeloid population My2 in s2.

**Figure 7:**
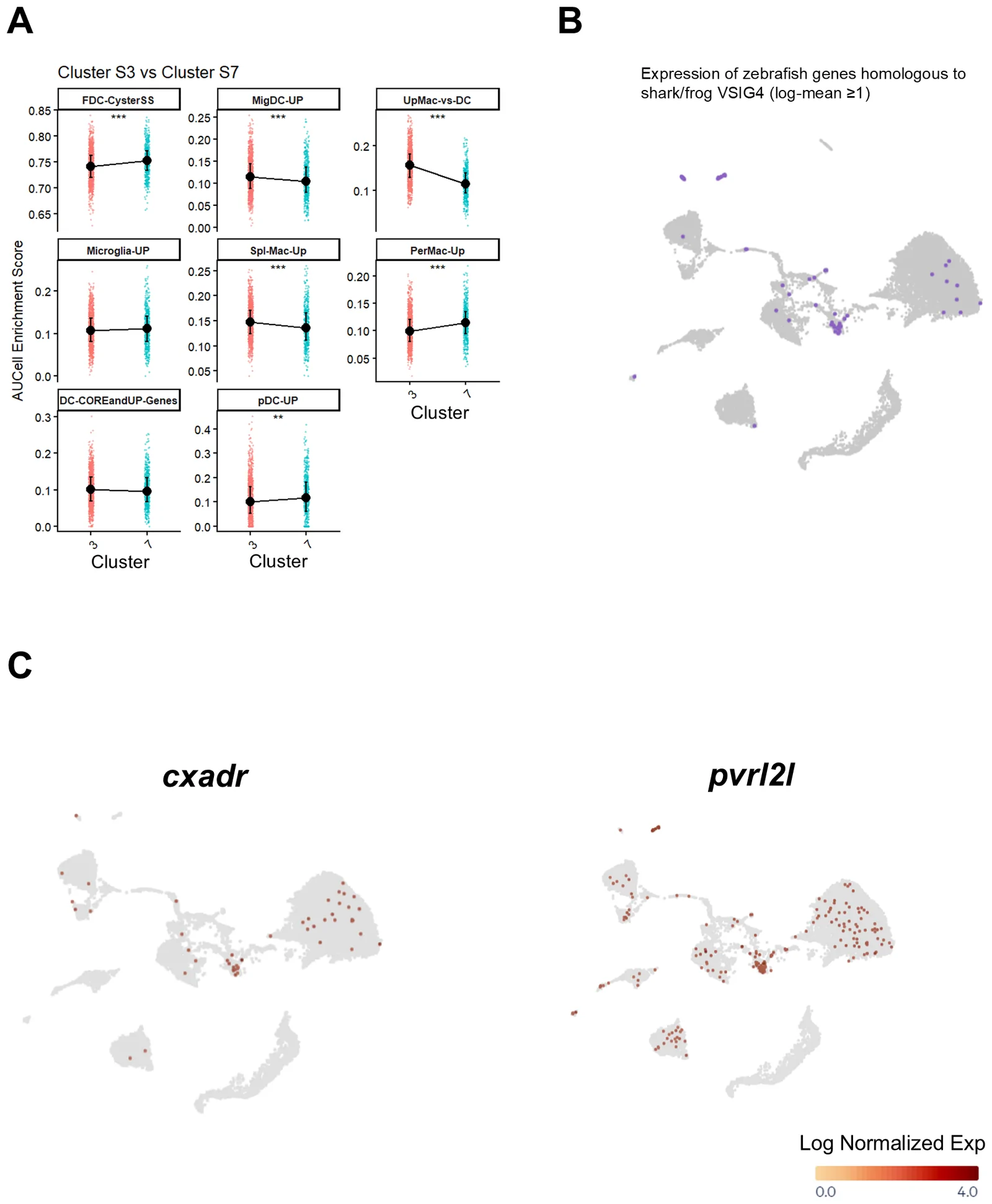
Zebrafish MMC melanomacrophage (S3) and pFRC-like (S7) signatures map to conserved myeloid and stromal lineages across vertebrates. **(A)** S3 shows enrichment of macrophage-associated gene sets, whereas S7 shows enrichment of FDC and pDC signatures when compared to mammalian immune gene sets using pairwise AUCell enrichment analysis. Median enrichment scores are shown as black dots per cluster with statistical testing performed using Wilcoxon rank-sum tests with Benjamini-Hochberg correction. **(B)** Zebrafish genes homologous to shark and frog VSIG4 showed the highest average log-normalized expression in S7. **(C)** UMAP feature plots show the highest expression of *cxadr* (putative Ig Fc receptor) and *pvrl2l* (*cd155*-ortholog) in S7.

To further resolve the vertebrate immune identities of MMs and pFRCs, we adopted the comparative strategy used in the *Xenopus* FDC-analogue study (51). We projected mammalian immune gene sets onto zebrafish single-cell data following orthology mapping. Across gene sets, 54% to 85% of mouse genes retained expressed zebrafish orthologs (**Supplementary Table 5**). We observed highest retention for macrophage-biased gene sets (UpMac_vs_DC, 84.8%), followed by FDC (FDC_CysterSS, 76.3%) and pDC (pDC_UP, 73.3%). Other gene sets showed intermediate conservation, including Spl_Mac_Up (61.9%) and PerMac_Up (61.7%) alongside MigDC_UP (58.6%), whereas the lineage-restricted Microglia_UP signature showed lowest retention (54%). We then calculated per-cell enrichment scores of these gene sets and summarized them at the cluster level. We found that S3 was preferentially enriched for dendritic cell genes selectively upregulated in CD103^+^ DCs (MigDC_UP), splenic macrophage genes (Spl_Mac_Up), and macrophage-biased programs (UpMac_vs_DC) (**Figure 7a**). In contrast, S7 preferentially enriched FDC (FDC_CysterSS) and pDC (pDC_UP) signatures (**Figure 7A**).

To investigate complement receptors implicated in antigen capture within GC-analogous structures, we examined conservation of VSIG4, highlighted in both shark and amphibian studies. We noted that the shark spleen atlas reported strong VSIG4-associated expression in My2 cells, while the *Xenopus laevis* study identified VSIG4 as the principal complement receptor mediating immune complex capture. Using PSI-BLAST with whale shark and *Xenopus* VSIG4 sequences, we recovered zebrafish butyrophilin and butyrophilin-like, as well as CXADR-like immunoglobulin superfamily proteins (E-value < 0.005) as closest shared relatives (**Supplementary Table 6**) with pFRCs showing the highest average expression of these genes (**Figure 7B**). Additional candidates included *vsig8a*, *pvrl2l*, *igsf11*, *mcam*, *cd22-like*, and siglec-like genes (**Supplementary Table 3**). Notably, *cxadr*, recently described as a human IgG Fc receptor (92), and *pvrl2l*, an ortholog of the FDC-associated receptor PVR (CD155) (93–96), were predominantly expressed in S7 (**Figure 7C**).

Collectively, these analyses indicate that the S3 and S7 populations share transcriptional features with vertebrate myeloid and stromal cell populations associated with GC-analogous structures.

## 4 Discussion

We sought to identify the cell types involved in affinity maturation in zebrafish and to propose the mechanisms underlying this process. Using single-cell RNA sequencing and marker-based annotation of zebrafish MMC cell clusters, we characterized both immune and non-immune cell populations. Based on marker and pathway enrichment analyses and guided by hallmarks of the mammalian germinal center reaction, we propose a model in which pFRCs and/or MMs mediate affinity maturation in zebrafish.

### 4.1 B-cell populations within the zebrafish MMC

In the canonical mammalian GC reaction, B cells transition between two main states: aicda^hi^cxcr4^hi^cd83^lo^cd86^lo^ centroblasts in the dark zone undergo clonal expansion and somatic hypermutation, while cxcr5^hi^cxcr4^lo^cd83^hi^cd86^hi^mhcII^hi^ centrocytes in the light zone undergo affinity selection (11, 97, 98). More recently, a three-zone GC model (52) subdivides the dark zone into a proliferation zone (DZp), where *cd83^+^cxcr4^+^* B cells proliferate or undergo apoptosis near tingible-body macrophages, and a differentiation zone (DZd), where *cd83^-^cxcr4^+^* B cells undergo AID-mediated somatic hypermutation.

We identified two B cell populations. Cluster S14 expressed markers including *pax5, cd81a, vav2, cd40*, and *cd99*, consistent with memory B cells. S10, however, showed a mixed profile. Its *mhc2* and *cd83* expression suggested a centrocyte-like identity under the canonical GC model. However, under the three-zone model, the *cd83^+^cxcr4^+^* signature aligned more closely with DZp centroblasts. Additional features supported this interpretation: high *myc*, which primes light-zone B cells for proliferation, and increased peptide-MHCII densities, reported in B cells recirculating from the light to the dark zone (11, 99). STRING enrichment further revealed both BCR-activation pathways typical of centrocytes and translational upregulation characteristic of centroblasts. Plasmablast identity was ruled out by lack of expression of BLIMP-1, a differential marker of B-cells committing to the plasmablast pathway. In line with this observation, S10 cells expressed Pax-5, the antagonist of BLIMP-1, and marker of germinal center fate in mammals (53). Together, these findings suggest that S10 represents proliferating centroblast-like cells that have undergone prior selection, leading to upregulation of *myc* and *mhc2*.

Having identified S10 as proliferating centroblast-like cells that have undergone selection, the question arises as to where somatic hypermutation (SHM) of the VDJ exons occurs. In catfish, *aicda*+ cells labelled by *in-situ* hybridization were visualized within or near the autofluorescent MMC (27), whereas in rainbow trout, SHM predominantly occurs in an adjacent aicda^+^ B cell-rich region that, together with the MMC and a neighboring T cell-rich zone, forms the MMC-associated lymphoid aggregate (M-LA) (39). Notably, trout lymphoreticular tissues contain MMCs that can be totally encapsulated, partially encapsulated, or free-moving (100), which may permit spatial segregation of B-cell expansion and SHM outside the MMC. In contrast, cyprinid (zebrafish and goldfish) and catfish MMCs are encapsulated by reticular cells (27, 30, 38), and aicda expression is predominantly localized to the MMC (27) and not to adjacent lymphoid-rich regions, suggesting that proliferation and selection may occur within the MMC itself. The limited detection of aicda transcripts in our dataset prevents us from directly demonstrating that somatic hypermutation was actively occurring within the captured MMC-associated B-cell populations at the time of sampling. This likely reflects several non-mutually exclusive factors: (i) splenic MMCs can occasionally lack detectable aicda expression even when SHM is ongoing, as previously reported (27); (ii) the absence of infection or strong immunization in our study, as insufficient stimulation has been shown to fail to induce aicda transcription in teleost B-cell systems (27); and (iii) limited sensitivity of scRNA-seq to detect cells expressing low levels of aicda transcripts. Future scRNA-seq studies following immunization should capture aicda expression.

### 4.2 T-cell populations associated with the zebrafish MMC

In the mammalian GC, high-affinity centrocytes escape apoptosis by receiving survival signals from T follicular helper (Tfh) cells, including CD40L-CD40 interactions and cytokines such as IL-4 and IL-21 (101–103). In our dataset, one T-cell cluster (S2b) expressed *cd4-1*, indicating a CD4^+^ lineage, along with high levels of *IL4* and *IL21* transcripts, consistent with Tfh-mediated survival signaling. Prior work on CCR9^+^ Tfh cells (104), which produce IL-21 to support B-cell survival, further supports this interpretation. S2b also expressed *tc7* and *cd28*, markers of T-cell development, as well as *gata3*, the Th2 lineage-defining factor, suggesting it may represent a naïve population differentiating toward Th2. While our data cannot confirm this trajectory, we propose that S2b functions as a Tfh analogue, providing survival signals that enable high-affinity B cells to avoid apoptosis and differentiate into plasma and memory cells.

In addition to Tfh-like cells (S2b), we also identified a CD8+ T-cell cluster (S12). These cells are reminiscent of cxcr5+ Tfc cells described first by Yu and Ye (105). Tfc cells have been shown to be functionally close to cytotoxic CD8+ cells, albeit experiencing less “exhaustion” than their typical CD8+ cytotoxic counterparts. This is in part due to their being closer to a memory-type rather than an effector-type phenotype. This differentiation was found to be positively regulated by Tcf1, Bcl6, and Id3 expression and negatively regulated by Blimp1 and Id2. Tfc cells were also characterized by low Tim-3 expression. Further indicative of the memory phenotype and lower “exhaustion” aligned with these findings, the S12 Tfc-like cells from our dataset expressed differentially high levels of Tcf1, Bcl6, and Id3, and low levels of Blimp1, Id2 and Tim-3. In the context of antibody affinity maturation in zebrafish MMC, we speculate that these Tfc-like cells, akin to cxcr5+ Tfcs in mammals, can have both positive regulation of antibody affinity maturation via interactions with B-cells and negative regulation via inhibition of Tfh cells. Thus, Tfc-like cells could be regulating the antibody affinity modification process in zebrafish MMCs.

### 4.3 Melanomacrophages and pFRC-like cells as candidate antigen-retaining populations

In the mammalian germinal center, FDCs display antigens as immune complexes (Ig: Ag), either directly on the cell surface (106) or as iccosomes (5). Antigen display can be sustained for up to a year through alternating cycles of internalization and re-exposure that protect immune complexes from degradation. High-affinity B cells bind these antigens, internalize and process them, and then present peptides to cognate Tfh cells, which in turn provide survival signals that prevent apoptosis (101, 103). Based on marker expression and antigen-presentation potential, we propose that two zebrafish populations, MMs (cluster S3) and pFRC-like (cluster S7b), serve complementary roles analogous to mammalian FDCs in supporting B cell selection within MMCs.

We identified MMs by *csf1ra* expression (27) and enrichment of iron-processing pathways, including ferroptosis, which contributes to lipofuscin accumulation, the histological marker of these cells, as reviewed by Wolke (107). MMs are hypothesized to be derived from hematopoietic stem cells (108). Aligning with this, we observed enrichment of HSPCs within MMCs, with cluster S0 preferentially localized to MMC regions compared to the whole spleen dataset. This suggests a local progenitor-supportive microenvironment within the MMC. MMs expressed high levels of *MARCO* and *c1q*, genes associated with mammalian marginal zone macrophages (MZMs) (109). As shown in mouse, MARCO, a broad-range receptor, has been shown to detach from MZMs and migrate along conduits to the follicle, where it anchors to the FDC membrane either directly or as part of a CD21-C4:MARCO complex. B cells can then recognize antigens presented on FDC-bound MARCO, providing a mechanism for affinity selection (109). On the other hand, MMs expressed high levels of MHC class II genes and other genes associated with phagocytosis, suggesting they may preferentially phagocytose and process antigens for presentation to CD4^+^ T cells rather than retain intact antigen on their surface. Their phagocytic phenotype further suggests roles in clearing pathogens and apoptotic B cells that fail selection. Nevertheless, it remains possible that zebrafish MMs or a specialized subset also retain intact antigen while performing these complementary functions.

Our data indicate that multiple MMC cell types in the zebrafish spleen originate from the perivascular niche. In mouse, this niche gives rise to reticular cells (81, 82). In zebrafish, reticular cells encapsulate MMCs (30). To explore this link further, we examined the expression of niche-associated markers including *anxa1a*, *cd9a*, *angpt1*, *ebf1a*, *cav1*, and *cav2 (*83). Three clusters (S4, S6, and S7) showed differential expression of these markers, supporting the contribution of perivascular-derived cells to the MMC and suggesting that they may play a role in zebrafish affinity maturation. We identified thrombocytes (cluster S4) based on the expression of canonical markers, including *hbegfb*, *thbs1*, *apln*, and *mpl* (41, 110, 111). Although not strictly perivascular, we speculate that their expression of perivascular markers reflects their origin from megakaryocytes, which shed thrombocytes into the sinusoids of the perivascular niche (112). Cluster S6 resolved into three populations comprising macrophage-like VSMCs (mlVSMCs), endothelial cells, and contractile VSMCs (cVSMCs) (83, 85). Unlike myeloid-derived macrophages, mlVSMCs participate in pro-inflammatory responses, whereas cVSMCs regulate vessel diameter through contraction (113). Endothelial cells, as expected, make up the lining of blood vessels. Notably, doublet analysis revealed that the top 4 pairings (80/110 doublets) all involved S6, being paired with S3 (Melanomacrophages), S4 (Thrombocytes), S10 (Centroblast-like) and S2 (Tc1 and Tfh-like cells). The pairing of S6 with each of these other cell types suggests that the 2 cell types constituting each pairing might have been interacting or exchanging cytoplasmic products at the time of sequencing library preparation. Although doublets are typically considered technical artifacts arising from cell contamination, the repeated involvement of S6 in the dominant doublet pairings suggests non-random association. Consistent with this, S6 represents blood vessel-associated cells, comprising endothelial cells together with vascular smooth muscle cell (VSMC) populations of the splenic vasculature, which contains abundant circulating erythrocytes and thrombocytes. We therefore interpret these doublets as reflecting physical proximity of vascular cells to MMC-associated immune populations at the time of tissue dissociation. MMs are found near specialized vasculature in the spleen called ellipsoid sheaths (114), and these were thought to be sites of antigen retention.

In contrast, cluster S7 emerged as the strongest candidate for an FDC-related population. S7 showed the highest cumulative expression of FDC-associated genes including *mfge8ab*, *clu*, *vcam1b*, *tnfsf13b*, *pdgfrb*, *c4b*, and *cxcl12a/b (*115, 116). As FDCs arise from ubiquitous perivascular precursors (preFDCs) expressing PDGFRβ, the remarkably high expression of *pdgfrb* supports interpreting S7 as an FDC precursor rather than a differentiated cell type (116). S7 expressed both perivascular niche and proteolytic enzyme genes. Reclustering resolved this population into two subclusters: S7a, identified as mast-like cells, and S7b, identified as progenitor fibroblastic reticular cell (pFRC)-like cells. Proteolytic enzyme genes were largely confined to S7a, whereas FDC-associated genes were predominantly expressed in S7b, refining the FDC analogue to this subcluster. Based on these features, we classified S7b as pFRCs, a population that includes both multipotent and intermediate perivascular reticular cells, distinguished by the absence of differentiation markers such as *bst1*, *enpp2*, and *clu*. In mammals, pFRCs differentiate into FRCs through LTβR-dependent signaling, with further cues driving commitment into specific subtypes such as MRCs, FDCs, or TRCs (117). Since the teleost immune system lacks the LT/LTβR pathway (91), this process may be arrested at the pFRC stage. Along with the strong expression of perivascular niche-associated markers in S7b, these findings support its identification as a perivascular pFRC population and the most likely zebrafish analogue of mammalian FDCs.

Additionally, the complement component C3, which is expressed by FDC in mammals and is known to contribute to long-term antigen retention on the surface of FDCs (90), had the highest expression in cluster S7 (pFRC-like). This reinforces the speculation that S7 is important in long-term antigen retention within the MMC. However, there are two caveats to this interpretation. In mammals, pFRCs are precursors to mature FDCs and are not themselves capable of antigen retention; only after LTβR and TNF family signaling do they differentiate into FDCs competent for immune complex display (81). In zebrafish, where the LTβR pathway is absent (91), we infer that pFRCs represent the terminal differentiation state of reticular cells, thereby potentially functioning as the FDC analogue. Second, although S7 expresses the highest cumulative set of FDC-associated markers, it lacks CD21/35, which are critical for immune complex presentation by mammalian FDCs (5, 117). It is likely, however, that zebrafish use alternative receptors to fulfill these roles, as complement receptors remain undefined and fish do not make IgG. Notably, ZRC1 and ZRC2 genes, which have been shown to encode members of the zebrafish Regulators of complement activation (RCA), could be ancestral versions of CD21 and CD35 genes. This is suggested by the conserved synteny of CD21 and CD35 genes in mammals with ZRC1 and ZRC2 genes in zebrafish (118). This is further supported by the phylogenetic branching of ZRC1 (alternatively named Tecrem in this study) from the same root as group 2 RCA molecules (119). Also, S7 shows strong expression of integrins and adhesion-associated genes, which have been implicated in B-cell selection by FDCs (87, 120). The recycling of immune complexes by FDCs is known to be actin-dependent (5). Together, these features suggest that pFRC-like cells in zebrafish may mediate B-cell selection through a ZRC1/2-dependent and FcγRIIβ-independent pathway, potentially reinforced by integrin-mediated synapses involving VCAM and ICAM.

### 4.4 Chemokine expression in the zebrafish MMC

Our data shows that MMC organization is dominated by the CXCR4-CXCL12 axis. In the canonical GC model, zonal structure is maintained by CXCR4-CXCL12 and CXCR5-CXCL13 gradients (121), whereas the three-zone model (52) assigns CXCR4 a regulatory role in the shift from proliferation to differentiation and does not include CXCR5-CXCL13 (52, 121). In our dataset, *cxcr5* expression was sparse and *cxcl13* absent, suggesting that CXCR5-CXCL13 plays little to no role. This aligns with a recent study in teleost showing that carp spleen primarily uses *cxcr4b*, and not *cxcr4a* and *cxcr5*, as a homing agent (122). Still, the presence of B cells with signatures of both centroblasts and centrocytes indicates that MMC function cannot be reduced to a purely dark zone analogue. In teleosts, paralogs *cxcr4a/cxcr4b* and *cxcl12a/cxcl12b* arose through whole-genome duplication in ray-finned fish (123, 124). With cxcr4b as the main homing agent in teleost spleen, we focused on the *cxcr4b*-*cxcl12a* axis, noting highest cxcr4b expression in all T and B-cell subtypes and highest *cxcl12a* expression in endothelial-like and pFRC-like cells. This mirrors mammalian GCs, where reticular cells produce CXCL12 in the dark zone (125) and supports a model in which *cxcl12*-expressing pFRC-like cells and endothelial-like cells attract CXCR4^+^ B cells and T-cells to the MMC and mediate selection via antigen presentation. Additionally, we identified other chemokine pairs in our dataset, such as *ccr9a*-*ccl25b*, which appear to act between *ccr9a^+^* B cells and *ccl25b^+^* pFRC-like cells. These less prominent gradients may also contribute to cell migration dynamics in the fish MMC, alongside the dominant *cxcr4b*-*cxcl12a* axis.

### 4.5 A working model of the zebrafish MMC

In the context of previous studies (27, 38) showing clonal expansion, somatic hypermutation, and selection within or adjacent to the MMC, our findings provide additional support for the MMC as a functional analogue of the mammalian GC. Our analysis suggests that the putative Tfh and FDC counterparts within the MMC express transcriptional profiles more consistent with precursor than fully differentiated cell states. Specifically, the pFRC-like cells most closely resembled FDC, whereas the Th2-like cells most closely resembled Tfh. However, these findings should be interpreted cautiously, as cell identities were inferred from transcriptomic markers and may be influenced by incomplete annotation of the zebrafish genome and the functional divergence of duplicated genes following the teleost-specific genome duplication.

**Figure 8:**
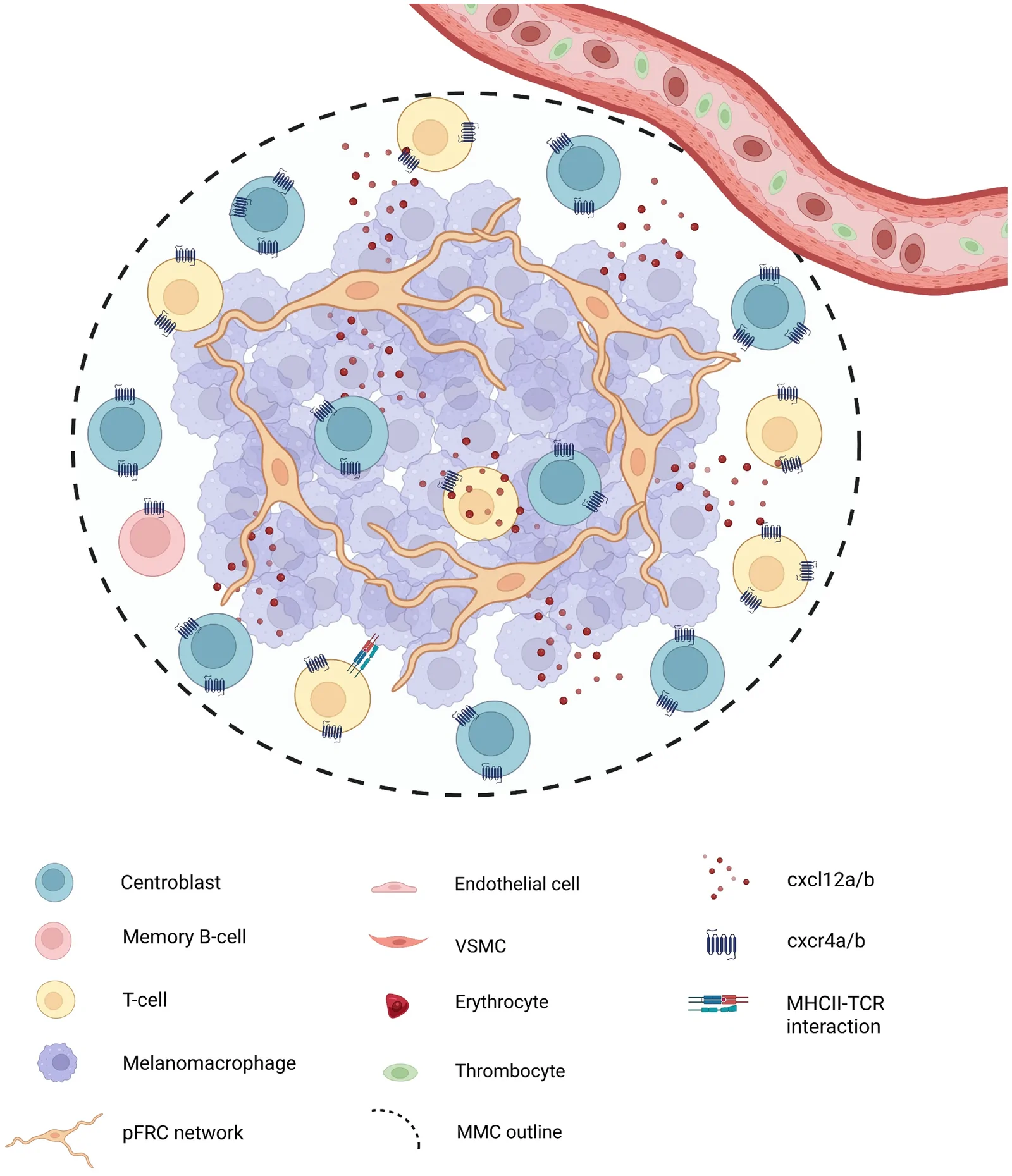
Proposed model of MMC-mediated antibody affinity maturation in zebrafish. CXCL12 is produced by pFRCs and recruits CXCR4+ B cells into MMCs. Inside MMCs, B cells proliferate and then undergo selection. Selection may involve antigen handling by pFRCs (S7) and/or melanomacrophages (S3). Like mammalian GC affinity maturation, Tfh-like (S2b) cells may provide survival signals in the MMC. (MMC, melanomacrophage center; pFRC, progenitor fibroblastic reticular cell; GC, germinal center).

We propose a working model (**Figure 8**) of zebrafish MMC-mediated affinity maturation in which the CXCR4-CXCL12 gradient attracts CXCR4^+^ B cells into the MMC, where CXCL12 is produced by pFRC-like cells (S7) and endothelial-like cells (S6). Within the MMC, B cells undergo proliferation, reflected by upregulation of translational and proliferative genes such as *myc*, and selection, potentially mediated by antigen trapped on pFRC-like cells and/or melanomacrophages. Antigen localization within MMCs observed previously (33, 126) may therefore reflect the activity of these cell types, though this requires confirmation. SHM is likely to occur between proliferation and selection, suggesting that SHM may require infection-induced stimulation and may not have been captured under the conditions examined here. In this model, selected B cells would internalize antigen, present it to Tfh-like cells, and receive survival signals. While the CXCR4-CXCL12 axis appears central, additional chemokine gradients, such as CCR9a-CCL25b, may also contribute. In contrast, the CXCR5-CXCL13 gradient seems less important in fish than in mammalian GC. Since scRNA-seq does not preserve the spatial organization of the whole spleen and its MMCs, future studies combining spatial, molecular, and functional approaches will be required to validate the cellular organization and interactions proposed here. Such studies will be important to establish the roles of the proposed immune and stromal cell populations in antigen retention, affinity maturation, and the organization of MMCs. In parallel, the development of additional antibodies against the markers identified in this study would enable protein-level characterization and functional validation of these cell populations.

### 4.6 Contextualizing the zebrafish MMC within existing models of vertebrate germinal center analogues

The model we propose adds to the existing frameworks of affinity maturation described in sharks (37) and frog (51) where antigen trapping within splenic follicles drives clonal selection. Both systems implicate VSIG4 as a complement receptor mediating immune complex capture, yet a clear VSIG4 ortholog could not be identified in zebrafish. Instead, we recovered CXADR as the closest homolog of VSIG4. CXADR was recently described (92) as an IgG Fc receptor. Although IgG is absent in teleosts and IgM is the dominant isotype, it remains possible that a CXADR-like receptor mediates IgM-based immune complex capture. Future studies using targeted inhibition or knockout of CXADR, together with other candidate receptors such as ZRC1, ZRC2, and MARCO, are needed to test their potential roles in antigen capture.

Previous work from Waly et al. (2022) (38) demonstrated several hallmarks of affinity maturation within zebrafish MMCs. Bulk VDJ repertoire sequencing of individual MMCs revealed that each MMC was dominated by a small number of expanded B-cell clonotypes containing hundreds of somatically mutated VDJ variants, alongside smaller clonotypes consistent with ongoing B-cell recruitment. Their mutation analyses showed an excess of replacement mutations within complementarity-determining regions relative to framework regions, consistent with antigen-driven positive selection. Though, their bulk VDJ repertoire data and single-cell marker analysis could be further extended using paired single-cell RNA-seq with full-length BCR/TCR sequencing following immunization to link expanded clonotypes, somatic mutations, and T-cell receptor repertoires to individual transcriptional states.

Waly et al. (2022) (38) showed that autofluorescent cells in zebrafish and goldfish retain intact antigen. These cells were presumed to be MMs based on their autofluorescence but were not characterized using additional cellular markers. For the scRNA-seq, the MMCs we dissected exhibited strong autofluorescence throughout, yet MMs comprised only ~8% of the recovered cells. This suggests that autofluorescence is not restricted to MMs and that additional cell types within the MMC also contribute to the overall fluorescent signal. We performed epifluorescence imaging of H&E-stained whole zebrafish spleen sections and found that green fluorescence emitted under blue-light excitation was not restricted to MMs, which were identified by their characteristic brown pigmentation. In addition to these pigmented MMs, several other splenic structures and cell types exhibited equally strong or even stronger green autofluorescence in some regions (**Supplementary Figure 3**). Furthermore, the intensity of brown pigmentation did not correlate with the intensity of green autofluorescence. The autofluorescence of non-MM cells and structures may have other cellular or extracellular sources. For example, collagen and elastin produced by stromal cells are known to autofluoresce in the blue/green spectral region (127). In our dataset, genes encoding these two are mostly produced by thrombocytes and the pFRC-like population. Recently, spectral flow cytometry revealed that human lymph node FRCs exhibit strong autofluorescence across ultraviolet to red channels, with yellow-green and red signals reaching up to 10^5^ (128). Additionally, erythrocytes in inflamed pig tissues have been shown to autofluoresce with a peak emission in the green region. Together, these suggest that autofluorescence may not be restricted to MMs and could be present in other MMC cell types.

We also showed that *csf1r* transcript expression is not specific to MMs and is elevated in other populations such as dendritic cells, VSMCs, pFRC, thrombocytes, and HSPCs/CEP. Furthermore, pFRC-like cells (S7b) are specifically enriched within the MMC and represent an MMC-associated population, whereas MMs are present both within the MMC and in surrounding splenic regions. Notably, pFRC-like cells express the highest cumulative set of FDC-associated genes including complement and adhesion molecules linked to antigen retention. This organization parallels the zebrafish axillary lymphoid organ (46, 129), where macrophages interact with a reticular stromal network, supporting another alternative model in which pFRC-like cells provide a structural scaffold while MMs hold the antigen, or both populations participate in antigen retention. In the absence of canonical complement receptors such as CD21/35, antigen retention may also involve alternative pathways including scavenger receptors such as MARCO (109), suggesting a non-canonical mechanism of immune complex handling in teleost MMCs.

Although fish MMCs have been implicated in antibody affinity maturation, the underlying mechanism has remained unclear. The zebrafish model we present integrates the hallmarks of the GC reaction with their putative cellular analogues in the MMC, providing a working framework for affinity maturation in teleosts.

## Data Availability

Single-cell RNA-seq data have been deposited in GEO under accession number GSE313637. Codes used in this study is accessible via GitHub at: https://github.com/rjlegaspi15/Cellular-composition-of-zebrafish-spleen-melanomacrophage-centers.

## Supplementary material

Supplementary Table 1 Summary of shared genes between zebrafish and shark datasets.

Supplementary Table 2 List of shared genes between zebrafish and shark datasets.

Supplementary Table 3 Enrichment of zebrafish S3 and S7 marker signatures across shark Seurat clusters.

Supplementary Table 4 Overlap of zebrafish S3 and S7 marker genes with shark lineage-defined cell populations across s1 and s2 datasets.

Supplementary Table 5 Summary of mouse gene set retention following orthology mapping to zebrafish genes.
