# Supplementary material for: Resolving the cellular profile of germinal center-analogous microstructures in zebrafish
Source: Front Immunol. 2026 Aug 31;17:1893026. doi: 10.3389/fimmu.2026.1893026 (PMC13572128; doi:10.3389/fimmu.2026.1893026)
Supplement: Supplementary file 1 [file DataSheet1.docx]

Supplementary Material

## 1 Supplementary Figures

**A**

**B**

**Supplementary Figure S1.** **Integration of whole zebrafish spleen and MMC scRNA-seq datasets**. **(A)** Bar graph showing the number of cells from MMC (blue) and PBS-injected spleen (gray) in each of the 27 clusters. **(B)** Mapping of the 27 integrated clusters onto the MMC UMAP (17 clusters) to assign cluster identities and visualize correspondence between datasets.

**A**

**B**

**Supplementary Figure S2.** **Identification and characterization of putative heterotypic doublets in zebrafish spleen MMCs**. **(A)** Bar plot showing the counts of predicted doublet pairs after lineage-agnostic scoring. Out of 110 heterotypic doublets, 101 have as S6 as a predicted pair. **(B)** Boxplots showing the distribution of the module scores of the top 1 and top 2 potential lineages of the doublets. Narrower gaps suggest two dominant transcriptional signatures likely arising from scRNA-seq artifact (co-encapsulation in the same gel bead) or incompletely disassociated tightly associated cell pairs. Wider gaps suggest ambient RNA contamination obscuring the single dominant signature

**Supplementary Figure S3. H&E and epifluorescence micrographs of non-infected zebrafish spleen sections.** Each spleen was dissected, after which it was fixed, stained with H&E and observed under both brightfield and the green channel of an epifluorescence microscope. The micrographs show that autofluorescence under the green channel is not restricted to melanomacrophages (arrows), which have been identified through their characteristic brown pigmentation. Other cell types (arrowheads) seem to autofluoresce at a higher intensity than the melanomacrophages in the green channel. Brightness and contrast were adjusted uniformly across all images in this panel. Scale bars are 20 μm. **(A)** Distinct area of spleen which appears to be made of connective tissue interspersed with brown pigmentation. The connective tissue area is surrounded by regions of circular cells with round nucleus which could be leukocytes or erythrocytes. **(B)** Distinct tissue which appears to be made up of connective fibers and elongated reticular cells, interspersed with brown pigmentation. The connective tissue area is surrounded by regions of circular cells with round nucleus which could be leukocytes or erythrocytes. **(C)** Region of the spleen showing no brown pigmentation under H&E staining, but still displays cells or tissues that autofluoresce green **(D)** Aggregation of large granulated cells and elongated reticular cells, interspersed with brown pigmentation.
